# Angiosarcoma in previously irradiated breast in patient with Li-Fraumeni syndrome. A case report: Angiossarcoma em mama previamente irradiada em paciente portadora da síndrome de Li-Fraumeni. Um relato de caso

**DOI:** 10.1590/1516-3180.2012.6740004

**Published:** 2014-09-26

**Authors:** Oséias Vargas Barbosa, André Borba Reiriz, Ricardo Antônio Boff, Willian Passos Oliveira, Luiza Rossi

**Affiliations:** I Medical Student. Faculdade de Medicina da Universidade de Caxias do Sul (FMUCS), Caxias do Sul, Rio Grande do Sul, Brazil.; II MD, PhD. Titular Professor and Head, Department of Oncology, Faculdade de Medicina da Universidade de Caxias do Sul (FMUCS), Caxias do Sul, Rio Grande do Sul, Brazil.; III MD. Mastologist, Faculdade de Medicina da Universidade de Caxias do Sul (FMUCS), Caxias do Sul, Rio Grande do Sul, Brazil.

**Keywords:** Radiotherapy, Hemangiosarcoma, Li-Fraumeni syndrome, Breast neoplasms, Genes, p53

## Abstract

**CONTEXT:**

Li-Fraumeni syndrome is a rare disease with an autosomal dominant inheritance pattern and high penetrance that defines a 50% chance of developing cancer before the age of 30 years, including cases of breast sarcoma. Patients with this syndrome who require radiotherapy have an increased risk of developing secondary malignancies including angiosarcomas.

**CASE REPORT:**

This was a case report on a female patient with Li-Fraumeni syndrome. In October 2005, she was diagnosed with invasive ductal carcinoma of the right breast and underwent sectorectomy. She then received chemotherapy and adjuvant radiotherapy. Trastuzumab and tamoxifen were also part of the treatment. She recently sought care at our hospital, complaining of hyperemia and nodulation in the right breast, and underwent surgical resection that revealed epithelioid angiosarcoma.

**CONCLUSIONS:**

When genetic predisposition due to Li-Fraumeni syndrome is documented, the therapy should be adapted so as to minimize the risk. Thus, conservative surgical treatments should be avoided and mastectomy without radiation should be prioritized. In cases in which use of radiotherapy is justified, patients should be followed up intensively.

## RESUMO

**CONTEXTO:**

A síndrome de Li-Fraumeni é doença rara que apresenta padrão de herança autossômica dominante e alta penetrância, definindo possibilidade de 50% no desenvolvimento de neoplasias antes dos 30 anos, incluindo nesses casos os sarcomas em mama. Pacientes portadoras dessa síndrome que requerem tratamento radioterápico têm risco aumentado de desenvolver neoplasias secundárias, incluindo os angiossarcomas.

**RELATO DE CASO:**

Este é um relato de caso de paciente feminina, portadora da síndrome de Li-Fraumeni. Em outubro de 2005, ela teve diagnóstico de carcinoma ductal invasor da mama direita, sendo submetida à setorectomia. Recebeu quimioterapia e radioterapia adjuvante; trastuzumabe e tamoxifeno também fizeram parte do tratamento. Recentemente, procurou atendimento em nosso serviço, com queixa de hiperemia e nodulação em mama direita, e foi submetida a ressecção cirúrgica que revelou angiossarcoma epitelioide.

**CONCLUSÕES:**

Quando a predisposição genética da síndrome de Li-Fraumeni está documentada, deve-se adequar a terapêutica a fim de minimizar riscos, evitando tratamentos cirúrgicos conservadores e priorizando a mastectomia sem radioterapia. Nos casos em que se justifica o uso de radioterapia, os pacientes devem ser acompanhados de forma intensiva.

## INTRODUCTION

Li-Fraumeni syndrome is an autosomal dominant disease and has high penetrance, thus defining a 50% chance of developing cancer before the age of 30 years. TP53 gene mutations that occur in Li-Fraumeni syndrome may predispose towards the rare diagnosis of sarcoma in breast cancer. Through current screening in young patients, increased prevalence has been diagnosed, thus leading to multimodal therapies including radiation, which has been recommended in cases of carcinoma. However, patients who undergo conservative therapy for breast cancer with surgery and adjuvant radiotherapy are at increased risk of developing secondary tumors in the breast.^1^ It is also known that radiotherapy is considered to be a risk factor for development of sarcomas in soft tissues, including breast tissue. Because of these factors, reports of malignancies secondary to radiotherapy have arisen, including cases of angiosarcomas.

## CASE REPORT

**Figure 1.**
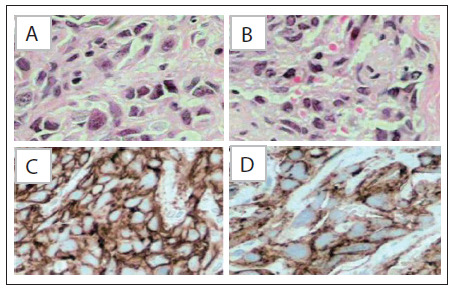
Neoplasm composed of spindle cells showing pleomorphic epithelioid eosinophilic cytoplasm and large hyperchromatic nuclei (A and B). Immunohistochemical study showing diffuse expression of CD 31 (C) and CD 34 (D).

The patient was a 32-year-old woman who had been diagnosed with adenocarcinoma of the sigmoid colon (pT3, N0, M0) in October 1999 and then underwent surgery followed by adjuvant chemotherapy with 5-fluorouracil and folinic acid. In October 2005, she was diagnosed with invasive ductal carcinoma of the right breast (pT2 N0 M0) and thus underwent sectorectomy and sentinel lymph node excision. She then received chemotherapy (anthracycline-based) and adjuvant radiotherapy. Trastuzumab and tamoxifen were also part of the treatment, given that she presented overexpression of Her-2 positive cells, estrogen receptors and progesterone. In June 2009, she was diagnosed with ductal carcinoma in situ and underwent mastectomy of the left breast. The patient underwent genetic counseling and the investigation revealed the gene mutation IVS6 + 1G > T in the gene TP53. Thus, in the light of her personal and family history, she presented the classical criteria for Li-Fraumeni syndrome. The patient recently sought care at our hospital, complaining of redness and nodulation in the right breast, and underwent surgical resection that revealed epithelioid angiosarcoma (Figure 1). Because of complete resection, she is under follow-up.

## DISCUSSION

The predisposition to neoplasms generated by Li-Fraumeni syndrome is clearly demonstrated in this case as we could observe in a systematic search of the literature, shown in Table 1. This syndrome causes a protein TP53 mutation that leads to inactivation of its repair mechanisms and apoptosis, which favors a cycle of cells in which the DNA has been damaged, thereby predisposing towards multiple cancers.^2^^,^^3^ Breast cancer generally occurs sporadically, but when it affects young people, it demonstrates greater possibility of heredity.^4^^,^^5^ With the advancement of genetic counseling and forms of early detection, some of these tumors have been diagnosed sooner. Thus, therapeutic methods become fully used, along with multimodal curative therapies.

**Table 1.**
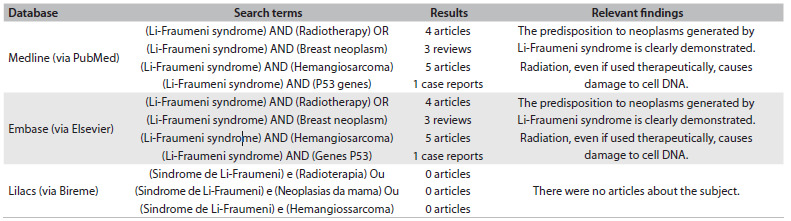
Search strategies performed on January 13, 2014, and results from PubMed, Lilacs and Emabse regarding the topic of Li-Fraumeni syndrome and radiotherapy and breast neoplasm and hemangiosarcoma or P53 genes

One of these methods is radiation, which plays a central role in adjuvant therapy for breast cancer. The benefits of adjuvant radiotherapy after breast-conserving surgery were demonstrated in a meta-analysis produced by the Early Breast Cancer Trialists’ Collaborative Group (EBCTCG).^6^ According to the study, radiotherapy reduces the risk of locoregional or systemic recurrence over a 10-year period from 35% to 19% (reduction of 15.7%), and at the age of 15 years, the risk of death is reduced from 25.5% to 21.4% (down by 3.8%).

However, it is known that radiation has been associated with predisposition towards some cancers.^4^^,^^7^^,^^8^^,^^9^ In addition, patients receiving radiotherapy for breast cancer remain at risk of secondary neoplasms and recurrence of the primary tumor for a long period after the treatment.^7^ Although rare, sarcomas constitute a well-documented complication of radiation therapy for breast cancer.^9^ They occur mostly after conservative therapies in the breast and chest wall, and also after mastectomy with or without radiotherapy.^7^^,^^10^ Angiosarcomas may occur in patients undergoing conservative breast therapies, as in the case presented here. This type of neoplasm has been studied and analyzed by many researchers, thereby confirming its association with adjuvant radiotherapy.^1^^,^^7^^,^^8^^,^^11^ The rationale for using radiation as an adjuvant treatment for breast cancer is that residual cancer cells might remain after the primary treatment. However, even if radiation is used therapeutically, it causes damage to cell DNA, as also shown in Table 1. Thus, it can be asked whether damage to genes that cause hereditary predisposition to breast cancer, as in Li-Fraumeni syndrome, might also affect radiosensitivity and favor the DNA damage caused by radiation. Therefore, radiation exposure, which alone constitutes a potential carcinogen, can be superimposed on preexisting susceptibility because of genetic syndromes. It is important to highlight that the risk of developing secondary malignancies through treatment is also higher in young patients, and that this is also linked to hereditary factors.^12^ Thus, we emphasize the need for appropriate therapy when disposition of this nature is documented, in order to minimize the risk and prioritize breast mastectomy without radiation. In cases in which use of conservative therapy and radiotherapy is justified, patients should be monitored intensively, taking into consideration the risk of developing secondary malignancies.

## CONCLUSIONS

The need for appropriate therapy when Li-Fraumeni syndrome is documented should be emphasized, in order to minimize the risk and prioritize breast mastectomy without radiation. Given the risk that patients with Li-Fraumeni syndrome might develop secondary malignancies when conservative therapy and radiotherapy are used, intensive monitoring in such cases is justified.
